# Population structure and emergence of resistance to new and repurposed drugs in XDR-TB: insights from a 10-year genomic study in the Western Cape, South Africa review

**DOI:** 10.3389/fcimb.2025.1638577

**Published:** 2025-09-04

**Authors:** Justice Tresor Ngom, Johannes Loubser, Elizna Maasdorp, Yonas Ghebrekristos, Sarishna Singh, Christoffel J. Opperman, Marisa Klopper, Robin Mark Warren, Elizabeth M. Streicher

**Affiliations:** ^1^ South African Medical Research Council Centre for Tuberculosis Research, Division of Molecular Biology and Human Genetics, Faculty of Medicine and Health Sciences, Stellenbosch University, Cape Town, South Africa; ^2^ South African Medical Research Council Centre for Tuberculosis Research, Division of Immunology, Faculty of Medicine and Health Sciences, Stellenbosch University, Cape Town, South Africa; ^3^ National Health Laboratory Service, Green Point TB-Laboratory, Cape Town, South Africa

**Keywords:** genomic characterisation, extensively drug-resistant tuberculosis, whole genome sequencing, transmission, new and repurposed drugs, South Africa

## Abstract

**Background:**

Extensively drug-resistant tuberculosis (XDR-TB) is a global health threat, being expensive and difficult to treat, with high mortality rates. The Western Cape Province (WCP), South Africa, has a particularly high burden of XDR-TB (>800 cases in the past ten years). Drug resistance genotypes and transmission present substantial regional variability. Thus, a better understanding of genetic diversity, clustering and the factors related to transmission can aid in prioritising resources to effectively target high-risk populations and regions that are disproportionately affected. We describe genetic diversity, drug resistance profiles and identify potential factors associated with the spread of XDR-TB strains collected in the WCP.

**Methods:**

We included 729 XDR-TB samples (one per patient), identified through routine diagnosis spanning 2010 to 2019, from six healthcare districts (HCDs) in the WCP. Genomic DNA from cultured isolates was sequenced using the Illumina platform. Sequences were analysed for strain type, drug resistance mutations, and genomic clustering using the TBProfiler and MTBseq pipelines. We conducted logistic regression analysis to identify potential factors associated with genomic traits related to the spread of XDR-TB strains.

**Results:**

Of the 729 XDR-TB strains, sublineage 2.2.2 (Atypical Beijing: n=378, 58.79%) strains were predominant, followed by Sublineage 2.2.1 (Typical Beijing: n=260, 40.43%). Atypical Beijing strains were more likely to cluster than Typical Beijing strains. Most of the clusters were small, with a few large and very large clusters, and the strains within very large clusters (primarily Atypical Beijing) were more likely to be found within Cape Town Metropole, Cape Winelands and Garden Route HCDs. Certain Atypical Beijing strains were found resistant to new and repurposed drugs recently introduced in the WHO treatment guidelines and clustered, indicating potential transmission.

**Conclusions:**

Near-untreatable Atypical Beijing strains are prevalent in the WCP. Hence, hotspot areas for clustering in Cape Town Metropole, Cape Winelands and Garden Route HCDs should be prioritised for targeted intervention to prevent ongoing XDR-TB transmission.

## Introduction

Extensively drug-resistant tuberculosis (XDR-TB) is a global health threat caused by *Mycobacterium tuberculosis* (*M.tb*). By the original definition, XDR-TB was multi-drug resistant (isoniazid and rifampicin resistance) with additional resistance to any fluoroquinolone and second-line injectable drugs (SLIDs). In 2021, XDR-TB was redefined to reflect newer regimens and ensure relevance; thus, rifampicin-resistant or multi-drug resistant (MDR) and two of the group A drugs – fluoroquinolones Moxifloxacin/Levofloxacin, linezolid, bedaquiline or clofazimine (World Health Organization Global Tuberculosis Report, 2023). XDR-TB is the most expensive and deadly form of drug-resistant tuberculosis (DR-TB). Diagnostic tests are not widely available and remain complex, while the treatment is difficult, long and requires highly toxic drugs, which are associated with poor outcomes (Zhang et al., 2021). South Africa has been at the forefront of introducing and scaling up new tools for the diagnosis and treatment regimens for DR-TB (Dheda et al., 2024).

Since the first XDR-TB outbreak occurred in 2006 in Tegula Ferry, South Africa (Gandhi et al., 2006), cases have been reported in 131 countries around the world (World Health Organization Global Tuberculosis Report, 2023), making the prevention of transmission critical. In 2022, 809 confirmed cases of XDR-TB were notified in South Africa, corresponding to 7.35% of all MDR/RR-TB and about 0.28% of all TB cases in the country (World Health Organization Global Tuberculosis Report, 2023). However, there exists considerable heterogeneity in drug resistance profiles and genotypes both within and between geographic regions (Chihota et al., 2012; Streicher et al., 2012b; Streicher et al., 2015; Chisompola et al., 2020), affecting the dynamics of drug resistance transmission across the country. Gaining insight into the mechanisms that contribute to the spread of XDR-TB and implementing measures to decrease transmission is crucial for the successful control of the DR-TB epidemic in the setting. Identification of potential factors may assist in allocating resources to specific groups of individuals and places that have a significant impact on transmission.

The Western Cape Province has one of the highest burdens of DR-TB in South Africa (Chihota et al., 2012; Streicher et al., 2012a; Said et al., 2023) and XDR-TB remains a particular concern (>800 cases in the past ten years). The decades-long spread of XDR-TB highlights that XDR-TB has become a critical concern in the province (Singh et al., 2007). This is alarming, as the province has significant population growth, partly due to the influx of migrant labourers and rapid urban and infrastructure development, and it is one of the world’s most popular tourist destinations (Western Cape Government). Previous studies conducted in the Western Cape Province reported population structure, the drug-resistance features and transmission of XDR-TB isolates (Chihota et al., 2012; Streicher et al., 2015; Oostvogels et al., 2022; Sy et al., 2022). However, few studies harness the full potential of whole genome sequencing to provide a genomic characterisation of XDR-TB strains present in the region.

We used Whole Genome Sequencing (WGS) of XDR-TB strains to describe the genetic diversity, identify the drug resistance features, define the genomic clusters, and identify potential factors associated with genomic traits related to the spread of XDR-TB. Since the study period is until 2019, we applied the old definition of XDR-TB, which would be relevant to our cohort at the time of treatment.

## Materials and methods

### Study population and setting

The whole genome sequences used in this study were obtained from stored isolates confirmed to be XDR-TB (old definition) through the routine diagnostic laboratories in the Western Cape Province (WCP) between 2010 and 2019. Routine diagnostic tests included culture-based DST (drug susceptibility testing), Cepheid GenXpert MTB/RIF, and line probe assay (GenoType^®^ MTBDR*plus* and MTBDR*sl*). The stored isolates were from the biobank housed at Stellenbosch University, Division of Molecular Biology and Human Genetics (MBHG). The MBHG biobank consists of all samples from the WCP that have been subjected to routine DST since 2007. During this timeframe, the biobank achieved an average sample recovery rate of 97.75% from the National Health Laboratory Service (NHLS).

Our study included 729 samples (one per patient) from the 1976 dataset isolates in the dataset. The sociodemographic (age and sex) and clinical data of patients (drug resistance profile and Acid-fast bacillus status), along with information regarding the location (healthcare district and subdistrict) and collection date, were abstracted from the NHLS database to investigate the association between variables. The study was approved by the University of Stellenbosch Health Research Ethics Committee (ethics reference number: N09-11-296).

### Bacterial culture and nucleic acid extraction

Stored isolates were revived in Mycobacterial Growth Indicator Tubes (MGIT) media with 0.8 ml of oleic acid-albumin-dextrose-catalase (OADC, BD Biosciences, San Diego, CA, 212240) growth supplement and incubated at 37°C in the BD BACTEC™ MGIT™ 960 culture system (Becton Dickinson Diagnostic Instrument Systems, Towson, Maryland, USA). Cultures were considered negative, where no growth was detected after 42 days of culture. Cultures that flagged positive before the cutoff were subcultured in Middlebrook 7H9 broth (Difco, Becton, Dickinson) supplemented with 2.5 ml of 20% Tween-80, 4 ml of 50% glycerol and 100 ml of OADC (7H9/Gly/TWEN/OADC) and incubated at 37°C. Strains from positive 7H9 culture were subjected to deoxyribonucleic acid (DNA) extraction using the standard phenol-chloroform (CTAB; Hexadecyltrimethylammonium bromide) and sodium dodecyl sulphate (SDS) method as described by van Soolingen et al., 1991 (Kohl et al., 2018).

### Whole Genome Sequencing

After DNA extraction, purity, integrity and quantity control, 50 µl aliquots (concentration > 20 ng/µl) of DNA were subjected to WGS (**Supplementary Methods**) using the Illumina Novaseq 6000 platform (2 x 150 bp configuration).

Whole genome sequences were used to determine the drug resistance profile and lineages using TBProfiler version 6.4.1 (Phelan et al., 2019) and to identify groups of related isolates with a comparative analysis using MTBseq v.1.1.0 (Kohl et al., 2018). Raw data in the form of FASTQ files were submitted to the ENA sequence read archive (**Supplementary Document I**; project accession numbers PRJEB43283 (Oostvogels et al., 2022) and PRJEB82658).

### Bioinformatics analysis

#### Genotypic drug resistance prediction and lineage assignments

TBProfiler version 6.4.1 was used to determine lineages and drug resistance variants, and to infer resistance profiles using the default pipeline parameters (Phelan et al., 2019). Variants were further reassessed with the mutation database ‘72ef6fa’ of the TBDB repository (https://github.com/jodyphelan/tbdb/tree/who) comprising the World Health Organization (WHO) catalogue of molecular targets for *M.tb* complex resistance interpretation V2 (World Health Organization, 2021).

#### Clustering analysis of *M. tuberculosis* strains

We used the MTBseq pipeline for a comparative analysis of multiple strain samples to infer relatedness based on the pairwise distance of distinct SNP positions using the command line options “—step,” “TBgroup,” and “—distance.” We varied the option “—distance” to change cutoffs (0, 5 and 12) as per the definitions of direct, recent, or ongoing transmission, respectively (Kohl et al., 2018).

A genomic cluster was inferred if the genetic distance between at least two isolates fell below the defined SNP distance cutoff. Cluster size was defined by categorising cases (isolates) into three groups: 2–5 cases per cluster (small cluster), 6–25 cases per cluster (large cluster), and more than 25 cases (very large cluster). The boundaries of these categories were informed by observed cluster sizes.

#### Phylogenetic tree construction

We utilised the resulting multi-sequence alignment (MSA) file generated from the MTBseq analysis to construct phylogenetic trees using IQ-TREE2 v2.2.0.3. (**Supplementary Methods**).

### Statistical analysis

Descriptive statistics were conducted using standard methods to illustrate the number and proportion of clustered strains, non-clustered strains and cluster size distribution. Visualisation was facilitated using GraphPad Prism 10. Statistical analyses were performed using Stata/IC v18.1 (Stata Statistical Software, Release 18, College Station, TX, USA). A standard logistic regression model was performed to assess the associations between variables (**Supplementary Methods**). We investigated the potential association between dependent variables (cases belonging to molecular clusters and different categories of cluster sizes: small, large and very large) and independent variables such as age, gender, healthcare district, and genetic features (drug resistance profile, lineage, and sublineage). Statistical significance was defined as α < 0.05.

## Results

### Study sample set and characteristics of XDR-TB isolates

Our study samples comprised 729 XDR-TB isolates with sociodemographic data. One isolate was selected per case identified through the laboratory routine diagnostics of XDR-TB. Patient isolates mainly originated from the Cape Town Metropole (n=553, 75.86%) healthcare district (HCD), followed by the Cape Winelands (n=66, 9.05%) and Garden Route (n=60, 8.23%) HCDs. Samples were collected from almost an equal number of male and female patients, with a sex ratio of 1.21. The age varied from 0 to 75, with a mean age of 35.55 years (SD=11.93), and the majority were young (**Table 1**). Acid-Fast Bacillus (AFB) smear status was unavailable for 17.28% of isolates. Of those with available smear status, 49.09% had negative AFB smear, and 50.91% had positive AFB smear (**Table 1**).

**Table 1:** General characteristics of the strains included in the study.

Characteristics	N	%
Sex		
Male	397	54.46
Female	328	44.99
Undeclared	4	0.55
Age		
<20 years	188	26.33
20–29 years	157	21.99
30–39 years	225	31.51
40–49 years	97	13.59
>= 50 years	47	6.58
Not provided	14	1.96
Years of sample collection		
2010	87	11.93
2011	82	11.25
2012	95	13.03
2013	75	10.29
2014	55	7.54
2015	111	15.23
2016	69	9.47
2017	66	9.05
2018	48	6.58
2019	41	5.62
Origin of samples (Districts)		
Cape Town Metropole	553	75.86
Cape Winelands	66	9.05
Garden Route	60	8.23
Overberg	14	1.92
West Coast	28	3.84
Central Karoo	1	0.14
Other Provinces	7	0.96
AFB smear results		
**<**9 (AFB/100 field)	43	5.90
1+ (10–99 AFBper100 field)	80	10.97
2+ (1–10 AFB/field)	54	7.41
3+ (>10 AFB/field)	130	17.83
Negative	296	40.60
Not provided	126	17.28

AFB, Acid-Fast Bacillus test.

### Population structure

#### Genetic diversity

Among the 729 sequenced isolates, we found three lineages: lineage 2 (n=643, 88.20%), lineage 4 (n=85, 11.66%) and lineage 1 (n=1, 0.14%). Lineage 2 strains were further assigned into three sublineages: L2.2.2 (Atypical Beijing family. n=378, 58.79%), sublineage L2.2.1 (Typical Beijing family. n=260, 40.43%) and sublineage L2.2.1.1 (n=5, 0.78%). Lineage 4 (L4) included eleven sublineages, of which sublineage L4.1.1.3 (n=40, 51.77%), L4.3.3 (n=10, 11.76%), and L4.3.2.1 (n=8, 9.41%) were most prevalent. The single Lineage 1 isolate was characterised as sublineage L1.2.2.2 and excluded from further analysis (**Supplementary Table S1**). In most HCDs, the predominant sublineage was L2.2.2, with L2.2.1 the second most dominant. However, in the West Coast HCD, lineage 4 was prevalent (**Supplementary Figure 1**).

#### Genotypic Drug Resistance profile

**Figure 1:**
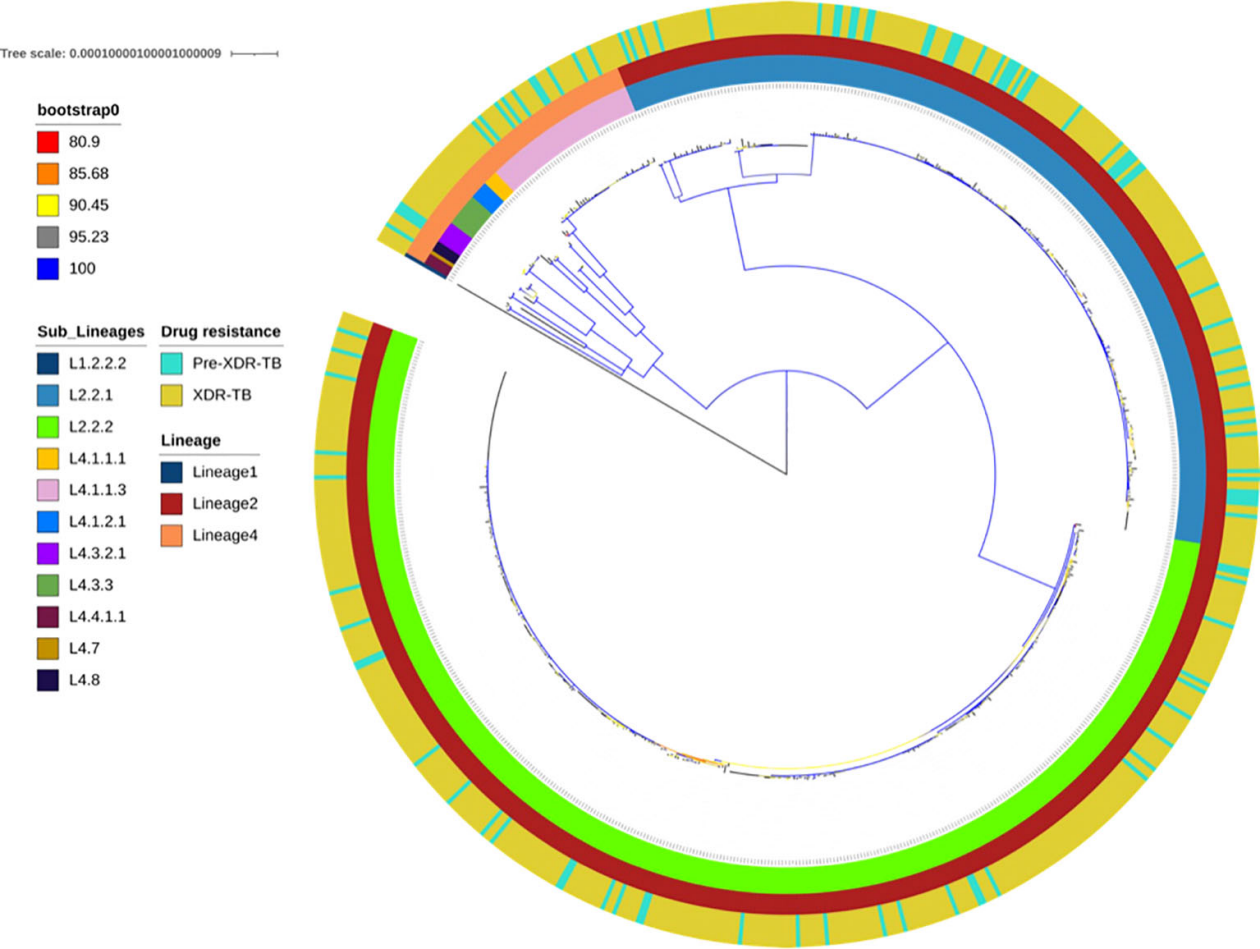
Maximum-likelihood phylogeny of 694 pre-XDR and XDR *Mycobacterium tuberculosis* isolates. The colour-coded annotation includes lineages, sublineages and drug-resistance profiles. The colour of the branches indicates the different degrees of bootstrap.

While all 729 isolates were confirmed as XDR-TB by routine laboratory diagnosis, only 590 isolates (80.93%) were found to be genotypically XDR-TB and 104 (4.26%) were pre-XDR-TB (multidrug-resistant isolate with additional resistance to fluoroquinolone or second-line injectable drug: SLID). The remaining isolates were described as Poly-DR-TB (n=5, 0.68%) and MDR-TB (n=23, 3.15%). There was no difference between the pre-XDR-TB population structure and the XDR-TB population structure (**Figure 1**). The proportional prevalence of XDR in each HCD was in accordance with the proportional general population size in each HCD, except possibly for CTM (**Supplementary Figure 2**; **Supplementary Table S2**). CTM seemed to carry a slightly larger than expected burden of XDR-TB. However, this may be explained by the much higher population density, as expected in urban vs. rural areas, which may facilitate transmission.

Resistance to additional drugs was observed in all the genotypically confirmed XDR-TB isolates (**Supplementary Table S3**). Ten isolates (1.69%) were additionally resistant to linezolid (LZD), and thirty isolates (5.08%) were cross-resistant to bedaquiline/clofazimine (BDQ/CFZ), with five isolates totally resistant (harbouring LZD and BDQ/CFZ resistance variants). **Supplementary Table S4** lists the drug resistance mutations observed in this study for 590 WGS-confirmed XDR-TB (specifying the mutations according to their lineages). A total of 30888 variants were detected, and only 18.13% (n=5601) were canonical variants associated with drug-resistance according to the 2023 WHO catalogue of mutations in MBTC and their association with drug resistance.

##### Rifampicin resistance variants

All the XDR-TB isolates harboured known variants that confer rifampicin resistance. The variants p.S450L (n=375/590; 63.56%) and p.D435V (n=176/590; 29.83%) were the most frequently observed within the *rpoB* gene. Both variants were observed alone or in conjunction with other *rpoB* variants (**Supplementary Table S4**). The p.S450L variant was found in strains across lineages (sublineage 2.2.1: n=192; sublineage 2.2.2: n=152; lineage 4: n=45), while the p.D435V variant occurred mostly in sublineage 2.2.2 strains (n=175).

#### Isoniazid resistance variants

Variants strongly associated with isoniazid resistance were identified within the *katG* (n=442/590; 74.92%), *fabG1 (inhA promoter)* (n=490/590; 83.05%), *inhA* (n=105/590; 17.79%) and *ahpC* (n=31/590; 5.25%) genes. The vast majority of isolates had variants in more than one of these regions (**Supplementary Table S4**).

The most prevalent *katG* variant was p.S315T, which was detected in 442 isolates (74.92%) and observed across lineages (sublineage 2.2.1: n=48; sublineage 2.2.2: n=335; lineage 4: n=59). In the *fabG1 (inhA promoter)*, the most commonly observed variants were c.-777C>T (n=305/590; 51.7%), mostly found in sublineage 2.2.1 and sublineage 2.2.2; followed by c.-779G>T (n=174/590; 29.49%) observed only in sublineage 2.2.2, and c.-770T>A (n=10/590; 1.69%) observed in sublineage 2.2.2 and lineage 4.

As for the *inhA* gene, the predominant variant was p.I194T (n=100/590; 16.94%), found only in combination with *inhA* promoter c.-777C>T in sublineage 2.2.1 isolates. Far less common, the p.S94A variant (n=3/490; 0.51%) was found solely in lineage 4 strains and always in conjunction with *katG* p.S315T. Only two other *inhA* gene variants were observed (one isolate each): p.I21T and p.I21V, in combination with additional INH resistance mutations.

The predominant *ahpC* variant was c.-48G>A, (n=28/590; 4.75%), while c.-57C>T was observed in three isolates. Both mutations occurred with *katG* p.S315T and were found exclusively in lineage 4 strains (n=31/590; 5.25%).

##### Fluoroquinolone resistance variants

Ten different variants were observed in the *gyrA* gene across all lineages (**Supplementary Table S4**), of which the most frequently observed variants were p.D94G (n=252/590; 42.71%), p.A90V (n=129/590; 21.86%), p.D94A (n=75/590; 12.71%) and pD94N (n=60/590; 10.16%), alone or in combination with additional *gyrA* and/or *gyrB* mutations. While 11 different *gyrB* variants were observed, the prevalent variants included p.E501D (n=10/590; 1.69%), p.R446C (n=8/590; 1.35%), and p.D461H (n=6/590; 1.35%).

#### Second line injectable drugs resistance variants

SLID resistance (amikacin: AMK, kanamycin: KAN, capreomycin: CAP) was caused mostly by four patterns of variants observed within the *eis* and *rrs* genes. Mutations in the *rrs* gene confer resistance to AMK, KAN, and CAP, mutations in the *eis* promoter confer resistance to AMK and KAN, while mutations in *tlyA* confer resistance only to CAP.

The most prevalent mutation was *rrs_*n.1401A>G, observed (AMK: n=555/590, 94.41%; KAN: n=561/590, 95.42%; CAP: n=552/590, 94.41%) across lineages (**Supplementary Table S4**). Five different *eis* promoter mutations were observed in a total of 36 isolates, three of which concurrently had an *rrs* mutation. Seven different *tlyA* variants were observed in individual isolates (three concurrently with *rrs*). The *tlyA* variants were all either insertions or resulted in premature stop codons.

#### Cycloserine resistance variants

Cycloserine resistance variants were observed within the *ald* and *alr* genes of 20.84% (n=123/590) of isolates (**Supplementary Table S4**). The *alr_p*.L113R was the most prevalent variant (n=111/590;18.81%) found mostly in sublineage 2.2.2. Only 9 isolates harboured *ald* mutations. The c.436_437dupGC variant alone was found in five sublineage 2.2.2 isolates, c.464delG alone was found in both sublineage 2.2.1 and Lineage 4, while a combination of these variants was found in a single Lineage 4 isolate.

##### Linezolid and bedaquiline/clofazimine resistance variants

Resistance variants were found within the *rplC* and *rrl* genes, contributing to LZD resistance and in the *mmpR5* and *pepQ genes*, resulting in CFZ/BDQ cross-resistance (**Supplementary Table S4**). The *rplC* _p.C154R (n=6/590; 1.02%) and *rrl* _n.2814G>T (n=4/590; 0.68%) variants were found only in sublineage 2.2.2 strains. All ten variants were classified as confirmed resistance-associated: tier 1 or Category 1 (**Supplementary Table S5**).

Within the *mmpR5* gene, the variants c144dupC (0.68%, N=4/590), pC46R (0.68%, n=4/590), p.Q115* (0.68%, n=4/590), c.139dupG (0.51%, n=3/590), c.198dupG (0.51%, n=3/590), and c.198delG (0.51%, n=3/590) was mostly observed in sublineage 2.2.2 strains. Only one isolate harboured the c.150_151dupCG variant in the *pepQ* gene. None of the *mmpR5* variants were found in the WHO catalogue of mutations V2 (**Supplementary Table S5**).

### Genomic clustering analysis

#### Clusters and size

After QC, a total of 582 sequences were eligible for analysis. Using the alternative cutoff values of 0, 5 and 12 SNP distances to define relatedness (in a particular transmission event), the proportions of strains that clustered were: 0 SNPs, 35.91% (209/582), 5 SNPs, 85,57% (498/582) and 12 SNPs, 97,59% (568/582) (**Supplementary Figure 3**). Further, while the largest cluster at 0 SNP distance was 8 isolates, increasing the threshold to 5 SNPs and 12 SNPs yielded cluster sizes of 149 and 171 isolates, respectively. In sublineage 2.2.1 and 2.2.2 each, two such large to very large clusters emerged at 5 and 12 SNPs distance cutoffs, while one large cluster emerged among lineage 4 isolates, only at the 12 SNPS distance cutoff. We proposed to present genomic clustering analysis at 0 SNP threshold here, while the remaining analytical results for the 5 SNP threshold are elaborated in the **Supplementary Document**.

**Figure 2:**
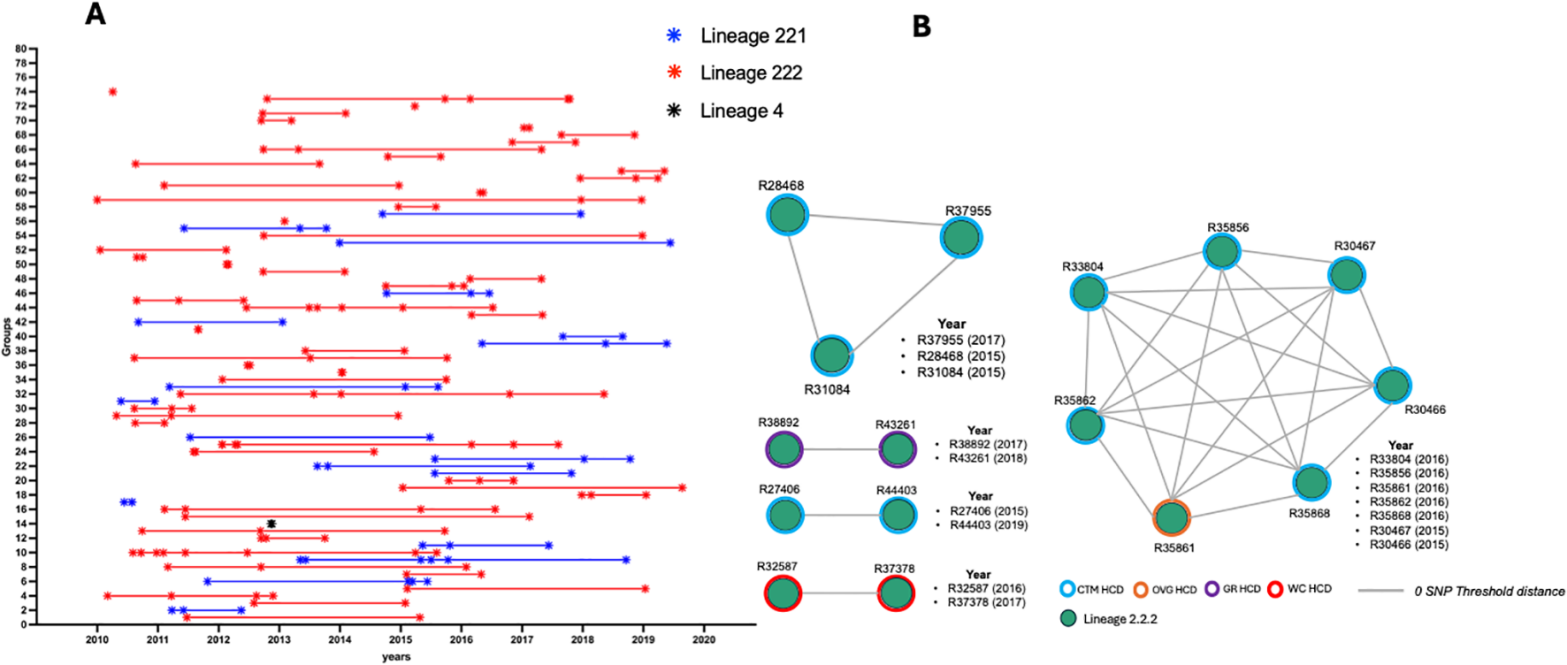
**(A)** Distribution of clusters over the study period and **(B)** clustering illustration of isolates harbouring new and repurposed drug resistance variants. HCD, Healthcare District; CTM, Cape Town Metropoles; OVG, Overberg; GR, Garden Route; WC, Winelands Cape.

The 0 SNP threshold (direct transmission) revealed that 74 genomic clusters had emerged, containing between 2 and 8 isolates per cluster. A cluster size of 2 was the most commonly found, with 39 groups of isolates, followed by 22 clusters comprising 3 isolates, 5 clusters comprising 4 isolates and 3 clusters comprising 5 isolates, respectively (**Supplementary Table S6**). Sublineage 2.2.2 strains formed more clusters than sublineage 2.2.1 and lineage 4 strains. The cluster distribution throughout the study period indicates that, in addition to the dominance of clusters formed with two isolates, most clusters were found within a period of three years or less (**Figure 2A**). Nearly half (15/34) of the isolates with new and repurposed drug-resistant variants were found within clusters, especially those showing LZD resistance variants (10/10). One cluster had four isolates harbouring LZD and BDQ/CFZ resistance variants simultaneously, while another cluster involved three isolates with resistance variants to LZD only (**Figure 2B**).

#### Logistic regression analysis

We performed logistic regression analyses on genomically clustered isolates (dependent variable, with unclustered isolates as reference category) at 0 SNP and 5 SNP distance thresholds and XDR-TB patient and strain characteristics (independent variables). The results are shown in **Supplementary Table S7**.

#### 0 SNP threshold

In the univariate analysis, the clustered isolates were found to be more likely to belong to sublineage 2.2.2 (OR=24.38; p<0.0001) and sublineage 2.2.1 (OR=10.47; p=0.001) compared to the unclustered group. These isolates were also more likely to infect patients aged between 20–29 years (OR=2.00; p=0.023) and 40–49 years (OR=2.26; p=0.01). Furthermore, these isolates were more likely to originate from patients living within Overberg (OR=17.14; p=0.016), Garden Route (OR=15.99; p=0.009), Cape Town Metropole (OR=14.41; P=0.009) and Cape Winelands (OR=10.58; p=0.027) HCDs.

In the multivariate analysis, clustered isolates were more likely to be found in patients aged between 40–49 years (OR=2.86; p=0.007), isolated in 2015 (OR=2.77; p=0.046), and belonging to sublineage 2.2.2 (OR=16.87; p<0.0001) and sublineage 2.2.1 (OR=8.95; p=0.006).

#### Genomic cluster size

Considering the 0 SNP threshold, in the multivariate analysis, isolates within small clusters were more likely to be isolated in 2013 (OR=27.23; p=0.004), 2015 (OR=11.91; p=0.02), and 2016 (OR=24.58; p=0.002) compared to isolates within the large clusters (**Supplementary Table S8**).

## Discussion

This research presents a genomic analysis of XDR-TB cases in the Western Cape Province (WCP), providing essential insights into genetic diversity and drug resistance profiles and potential factors related to the spread of XDR-TB strains over a decade (2010-2019). Our study revealed the presence of two primary lineages (lineage 2 and lineage 4) with sublineage 2.2.2 strains predominant, followed by sublineage 2.2.1. A discordance was found between routine laboratory methods and whole-genome sequencing (WGS) results, emphasising the necessity of integrating these two diagnostic strategies to identify resistance mutations accurately. We discovered isolates with variants that confer resistance to both new and repurposed drugs, underscoring the challenges associated with the implementation of the new treatments for XDR-TB patients. Genomic clustering analysis revealed several clusters where certain involved strains harbouring resistance variants to new and repurposed drugs. Logistic regression identified significant factors related to transmission, such as age, time period, and sublineage, with sublineage 2.2.2 demonstrating increased transmissibility and clustering tendency. Cluster sizes exhibited significant variation, influenced by time periods and geographic areas (healthcare districts). The findings highlight the necessity of incorporating whole genome sequencing (WGS) into standard surveillance practices and customizing interventions to combat the persistent transmission of extensively drug-resistant tuberculosis (XDR-TB) in WCP.

### Population structure

The population structure of XDR-TB isolates was dominated by lineage 2 (sublineage 2.2.2: Atypical Beijing and sublineage 2.2.1: Typical Beijing) and a few lineage 4 strains. Lineage 2 and lineage 4 are widely distributed in South Africa with diverse proportions, depending on geographic location (Chisompola et al., 2020). Previous studies have reported the dominance of lineage 2 and a smaller presence of lineage 4 strains in the WCP (Chihota et al., 2012; Streicher et al., 2015; Dheda et al., 2017; Oostvogels et al., 2022). This pattern was also evident in other provinces, including Eastern Cape, Gauteng, Limpopo, Mpumalanga, and North-West provinces (Said et al., 2012; Klopper et al., 2013). Moreover, the predominance of sublineage 2.2.2 in our cohort was also observed among XDR-TB cases in the Eastern Cape Province, known to be difficult to treat, with resistance to up to 14 drugs (Klopper et al., 2013). Conversely, lineage 4 was reported as the predominant strain in KwaZulu-Natal and Limpopo provinces, where lineage 2 is less prevalent (Maguga-Phasha et al., 2017; Yuengling et al., 2018). This underlines the distinct historical and geographical adaptation and spread of the two lineages within the country, particularly the WCP.

Both lineages have been documented to be hyper-mutable, giving them a propensity to develop resistance at a higher frequency than other lineages (Ford et al., 2013) and increasing overall genomic diversity within the lineage (Coll et al., 2018). Additionally, Studies from Peru and Thailand have indicated that lineage 2 has a higher risk of acquiring resistance than lineage 4 (Torres Ortiz et al., 2021), and presents a high degree of clustering, which could suggest more effective transmission of drug-resistant *M.tb* than other lineages (Nonghanphithak et al., 2021). In our study, lineage 4 comprised eleven sublineages compared to lineage 2 with three sublineages, suggesting that multiple unique strains evolved to XDR, with limited subsequent spread. The epidemiology of XDR-TB is shaped by both the ability of strains to gain resistance-causing mutation and their transmissibility—traits that are characteristic of lineage 2 and lineage 4 isolates, which may explain their higher prevalence compared to other lineages in the WCP. Active surveillance is essential to track their evolution and efficiently respond to a particular outbreak.

Notably, only a single case of lineage 1 (sublineage 1.2.2.2) was found in the entire cohort, which aligns with the diverse population structure of other regions, except for Mpumalanga and North-West (Said et al., 2012; Klopper et al., 2013). This lineage is more commonly seen in MDR-TB populations (Said et al., 2012), indicating it is less likely to evolve into XDR-TB.

### Discordance between routine laboratory methods and WGS

In our study, samples were collected and analysed for XDR-TB during a period when the older WHO definition of XDR-TB was used (Centers for Disease Control and Prevention (CDC), 2006). Almost all strains (95.20%) identified as XDR-TB through routine laboratory methods, including MGIT culture, GeneXpert, and Hain Line Probe Assay, were confirmed to be XDR through WGS. A few strains were described as poly-DR-TB and MDR-TB due to a lack of identifiable resistance markers to relevant drugs. This may be the result of unknown mechanisms of resistance, factors such as efflux pumps (resistance mechanism not based on single mutation), or selection of less-resistant subpopulations during culturing (Metcalfe et al., 2017). WGS can identify resistance to all TB drugs endorsed by WHO compared to routine laboratory methods. However, it is limited only to mutations known to be associated with drug resistance and still depends on culture that introduces the risk of missing some subpopulations with divergent drug-resistance phenotypes.

Our results underscore the importance of integrating WGS in routine laboratory methods to enhance drug resistance profiling of isolates and facilitate earlier individualised treatment. However, the method for performing WGS directly on sputum should be improved, and generating large-scale whole-genome datasets, coupled with associated minimum inhibition concentration (MIC) testing, is crucial to address disparities between results obtained through WGS and pDST. Accurate quantification of resistance levels associated with mutations is essential for optimising antitubercular medicines and selecting appropriate drugs and dosages in clinical situations (Rao et al., 2023). This approach may also assist in updating the WHO catalogue of mutations in the *Mycobacterium tuberculosis* complex and their association with drug resistance (World Health Organization, 2023).

### Variants conferring drug-resistance

The vast majority of isolates had canonical or high-confidence mutations conferring resistance to most or all drugs in the standard regimens used at the time (first line: INH, RIF, EMB, PZA; second line: AMK, KAN, CAP, FQ, CYC). Clonal spread of some of these variants was observed, largely driven by sublineage 2.2.2 transmission.

We found thirty-five isolates harbouring variants in genes associated with resistance to linezolid and bedaquiline/clofazimine. Bedaquiline and linezolid are key components of the BPaL/M (Bedaquiline, Pretomanid, Linezolid and Moxifloxacin) regimen newly approved by the World Health Organization for MDR, pre-XDR-TB and XDR-TB (old definition) treatment (World Health Organization, 2022). The resistance to these new and repurposed antituberculosis drugs has already been reported across four continents (Pang et al., 2017; Xu et al., 2017; Beckert et al., 2020; Wang et al., 2021; Yao et al., 2021; Ghodousi et al., 2022; Derendinger et al., 2023; Monde et al., 2023; Timm et al., 2023; Conkle-Gutierrez et al., 2024). Random mutations during evolution or medication exposure may explain resistance in these isolates. Linezolid was launched in 2000 to treat methicillin-resistant *Staphylococcus aureus* (MRSA) pneumonia and probable hospital- or ventilator-acquired pneumonia (Bamford et al., 2021) and was only used eleven years later as part of a multi-drug regimen to treat patients with XDR-TB or pre-XDR TB in the WCP (Medecin Sans Frontier, 2014). The use of bedaquiline in South Africa was approved by the Medicines Control Council in 2012 through the expanded Bedaquiline Clinical Access Programme (BCAP), where only 200 patients participated (Conradie et al., 2014). Unfortunately, data indicating participation in this programme was not available for our cohort. Bedaquiline and linezolid were introduced in standard regimens for pre-XDR-TB and XDR-TB patients in the 2015 South African guidelines (National Department of Health, 2015) and was extended to all centres of Excellence and made available at the district level as of 2017 (Pai et al., 2022).

The majority of variants (resistant to linezolid and bedaquiline/clofazimine) observed in our study is currently uncatalogued, and only a subset of these isolates were part of the transmission clusters. This suggests that resistance to components of the BPAL/M or BPAL/L regimens was uncommon prior to their programmatic introduction. These findings provide a promising baseline for these regimens in patients with MDR, pre-XDR-TB, and XDR-TB, despite reports of emerging resistance in certain countries and the risk of transmission (Goig et al., 2025). However, the presence of such variants in several isolates within certain genomic clusters, suggests transmission in addition to spontaneous emergence of resistance. The additional evolution of resistance markers to new and repurposed drugs underlines the severity of the situation in particular in sublineage 2.2.2 strains that were essentially untreatable (Klopper et al., 2013) before introduction of new drugs, and are losing new treatment options rapidly. Patient treatment informed by the combined use of phenotypic drug susceptibility testing and WGS or targeted sequencing is crucial to avoid the rapid emergence of resistant strains and their transmission across the region.

### Genomic clustering and associated factors

Our study examined factors influencing the spread of XDR-TB by analysing the characteristics of genomic clustered isolates compared to non-clustered isolates. Logistic regression analyses indicate that transmission of XDR-TB strains is influenced by factors such as time period, patient age, and sublineage, with sublineage 2.2.2 demonstrating increased transmissibility as exhibited by a large degree of clustering. Patients aged between 40 and 49 years, and the year 2015 were associated with an increased likelihood of direct transmission events (0 SNP distance) of XDR-TB, while the year 2018 was linked to higher likelihood of recent transmission events (5 SNP distance) of XDR-TB. Interestingly, these factors appear to be independent of the general population size regarding this age group or year. However, we cannot rule out a sampling bias that may have occurred in 2015 and 2018, potentially influencing these observations.

Our analysis revealed that sublineage 2.2.2 strains had 1.88 and 2.76 times higher likelihood to cause direct and recent transmission, respectively, compared to sublineage 2.2.1. This indicates that sublineage 2.2.2 strains may have gained certain advantages, such as increased transmissibility or clustering propensity, enabling them to spread more effectively than sublineage 2.2.1 strains in this region. This could suggest that the number of undiagnosed sublineage 2.2.2 strains is greater than that of sublineage 2.2.1. Alternatively, the mutation rate of sublineage 2.2.2 strains could vary from other lineages. Another likely scenario is that due to sublineage 2.2.2 being essentially untreatable, there was increased opportunity for transmission by failing to cure the infection. Further investigation into these potential mechanisms is warranted.

Many factors can influence the clustering mechanism of strains, including host-pathogen interaction, the strain genotypic background, treatability, transmissibility and the geographical setting, which often reflects human social behaviour and proximity to other regions where the strain is highly prevalent. Our findings align with other studies reporting that *M.tb* lineages and sublineages vary in their geographic distribution, likelihood of spread, and virulence (Coll et al., 2014; Stucki et al., 2016).

Additionally, the transmissibility of *M.tb* strains may be influenced by the fitness costs associated with particular resistance-conferring mutations (Gagneux et al., 2006). For instance, Human Immunodeficiency Virus (HIV) infection has been shown to be associated with low-fitness *rpoB* mutations in a multi-country study of patients with both negative and positive HIV status (Loiseau et al., 2020). Recently, a study using a phylodynamic approach found that on average, HIV-positive TB cases caused significantly fewer secondary TB cases compared to HIV-negative TB cases (Windels et al., 2024). Thus HIV coinfection, information that is unfortunately unavailable for this dataset, could significantly influence the transmission of specific strain lineages within our cohort. Further study should investigate the role of HIV coinfection in the emergence and transmission of *M. tuberculosis* XDR-TB sublineage 2.2.2 and sublineage 2.2.1 strains in the WCP.

### Cluster sizes and associated factors

We explored the various factors that influence the spread of XDR-TB in different cluster sizes (small, large and very large) by analysing cases according to genomic cluster size, cases and XDR-TB strain characteristics at the 0 and 5 SNP threshold distance. The findings revealed that the years 2013, 2015 and 2016 were linked to an increased likelihood of XDR-TB isolates spreading in small clusters with direct transmission (0 SNP distance) compared to large clusters. Regarding recent transmission (5 SNPs distance), the years 2010 and 2016 were linked to an increased likelihood of XDR-TB spreading in very large clusters, compared to other cluster sizes (large and small). The spike in direct or recent transmission events in the years 2010, 2013, 2015 and 2016 suggests that XDR-TB cases in these years may have been part of outbreaks where the strains spread quickly among close contacts or specific communities and within healthcare districts. These years could have been marked by environmental, socioeconomic and epidemiological conditions that promoted transmission, such as economic downturns, urbanization, limited TB care and control resources, and poor living conditions that might have contributed to the rapid spread of these XDR-TB strains within a defined population or geographic area. Potential late onset of disease and health care seeking behaviour complicates determining possible time-bounded causes of increased transmission.

Furthermore, the results revealed that sublineage 2.2.1 XDR-TB isolates were associated with an increased likelihood of spreading in large and small clusters compared to other cluster sizes (small and very large; large and very large) as recent transmission events (5 SNP).

Living within Cape Town Metropole, Cape Winelands and Garden Route HCDs increased the likelihood of XDR-TB strains spreading in very large clusters compared to other cluster sizes (small and large). Although Cape Town Metropole has the highest number of people infected with DR-TB (Sy et al., 2022), Garden Route is neighbouring the Eastern Cape Province, which is one of the highest-burden DR-TB regions in SA (National Department of Health, 2020), with a predominance of sublineage 2.2.2 strains that are difficult to treat (Klopper et al., 2013). Garden Route is between the Eastern Cape and Cape Town Metropole, and together with Cape Winelands, it represents the route used by migrants moving to Cape Town looking for jobs or good healthcare. In South Africa, some of the predominant Atypical Beijing strains originated from AA1- progenitor strains (sublineage 2.2.2 with a certain set of resistance markers) and were introduced from the Eastern Cape Province and then spread across the country (Klopper et al., 2020). Large clusters of prevalent genotypes can become established in some areas due to prolonged and uncontrolled transmission (Said et al., 2023). Community-based active case-finding interventions are important across the setting, particularly in those HCDs.

In our study, we used whole genome sequencing of a large sample of isolates collected over a period of ten years in order to understand the epidemiology of XDR-TB strains in the WCP and the associated factors. Our findings can assist the National Tuberculosis Program in prioritising targeted intervention. However, our study had some limitations. First, isolates included in the analyses do not represent all XDR-TB cases in the WCP over the ten-year period. Some cases may not have been diagnosed, and genotypic drug susceptibility tests were not carried out on sequences from all first-passage cultured isolates. Secondly, we used TB Profiler to detect high-confidence mutations and did not assess for heteroresistance. This may have resulted in the misclassification of some strains and could have affected our sample size and resistance profile. Thirdly, the SNP-based clustering method may contribute to unavoidable bias where strains could be misclassified as unique if they, in fact, clustered with strains outside the study period and geographical setting. Furthermore, this method of assigning clusters does not take into account variable mutation rates. The location where the XDR-TB isolates were collected might not represent the exact origin of the patient because of the mobility of people between infection and the development of symptoms. Lastly, the absence of clinical data (such as comorbidities, treatment history, previous laboratory results, etc.) or socioeconomic factors (e.g., access to healthcare, living conditions) could limit the scope and depth of our conclusions. Additionally, we were unable to do contact investigations to support the clusters and confirm the linkage between clustered isolates using epidemiologic data.

## Conclusions

XDR-TB has become a significant and persistent public health concern in the region over time, predominantly caused by difficult-to-treat sublineage 2.2.2 strains forming clusters, including very large clusters, spread across almost the entire province, but focussed in Garden Route, Cape Town Metropole and Cape Winelands HCDs. Our results suggest an adaptive advantage –likely the breadth of drug-resistance—of sublineage 2.2.2 strains, which spread more than sublineage 2.2.1 strains.

An alarming finding was that some sublineage 2.2.2 isolates, which are already resistant to all drugs in the old second-line regimen, were found resistant to new and repurposed drugs recently introduced in the treatment guidelines of the WHO. This urgently calls for increased antibiotic vigilance through pro-active case finding and targeted drug-susceptibility testing in high-risk regions. The introduction of WGS in routine laboratories will allow such precision medicine and molecular surveillance to enhance the efforts of XDR-TB care and prevention within a particularly high-burden setting, such as South Africa and the Western Cape.

The finding highlight the necessity of incorporating WGS into standard surveillance practices and customizing interventions to combat the persistent transmission of XDR-TB in WCP. However, real-time analysis including cluster analysis of WGS data is not yet feasible within high-TB burden settings such as South Africa.

## Data Availability

The datasets presented in this study can be found in online repositories. The names of the repository/repositories and accession number(s) can be found in the article/**Supplementary Material**.
