# Supplementary material for: Population structure and emergence of resistance to new and repurposed drugs in XDR-TB: insights from a 10-year genomic study in the Western Cape, South Africa review
Source: Front Cell Infect Microbiol. 2025 Sep 4;15:1638577. doi: 10.3389/fcimb.2025.1638577 (PMC12443792; doi:10.3389/fcimb.2025.1638577)
Supplement: Supplementary file 1 [file DataSheet1.docx]

**Population structure and emergence of resistance to new and repurposed drugs in XDR-TB: Insights from a 10-year genomic study in the Western Cape, South Africa review**

**Supplementary Materials and Method**

Justice Tresor Ngom^1*^, Johannes Loubser^1^, Elizna Maasdorp^2^ Yonas Ghebrekristos^1,3^, Sarishna Singh^1,3^, Christoffel J. Opperman^1,3^, Marisa Klopper^1^, Robin M. Warren^1^, Elizabeth M. Streicher^1^

**Affiliations**

1. South African Medical Research Council Centre for Tuberculosis Research, Division of Molecular Biology and Human Genetics, Faculty of Medicine and Health Sciences, Stellenbosch University, Cape Town, South Africa
2. South African Medical Research Council Centre for Tuberculosis Research, Division of Immunology, Faculty of Medicine and Health Sciences, Stellenbosch University, Cape Town, South Africa
3. National Health Laboratory Service, Green Point TB-Laboratory, Cape Town, South Africa

***Corresponding author**:

Justice Tresor Ngom

South African Medical Research Council Centre for Tuberculosis Research, Division of Molecular Biology and Human Genetics, Faculty of Medicine and Health Sciences, Stellenbosch University, Cape Town 7505, South Africa

Email: [justicengom@sun.ac.za](mailto:justicengom@sun.ac.za)

**Supplementary Methods**

*Determination of the isolates origine*

The information derived from the collection facility (NHLS). Each sample originated from a specific TB treatment and management facility, which were grouped into Healthcare District using WCG Department of Health open portal (<https://wcg-opendataportal-westerncapegov.hub.arcgis.com/datasets/westerncapegov::wcg-department-of-health-western-cape-health-sub-districts/explore>).

*Bacterial Culture*

From the original frozen stocks (stored at -80°C), isolates were inoculated in Mycobacteria Growth Indicator Tubes (MGIT) culture media supplemented with 0.8 ml of oleic acid-albumin-dextrose-catalase (OADC, BD Biosciences, San Diego, CA, 212240).. For each isolate, 2 or 3 glass beads coated with *M. tuberculosis* and stored at -80°C were removed from Nunc vials, under sterile conditions, and added to the supplemented MGIT tubes.. Each tube was incubated at 37°C in the BD BACTEC^TM^ MGIT^TM^ 960 culture system (Becton Dickinson Diagnostic Instrument Systems, Towson Maryland, USA) until a culture flagged either positive or negative (no growth were detected after 42 days of culture). Positive cultures were confirmed to be *M. tuberculosis* complex using the Capilia TB assay (TAUN, Numazu, Japan) and the Ziëhl-Neelsen (ZN) staining method. After the MGIT culture flagged positive, it was further incubated for 14 days prior to subculturing. DNA extraction cultures were prepared by inoculating 1 ml of positive MGIT culture into a 25 cm^2^ culture flask labelled (name, date and content) containing 5 ml of Middlebrook 7H9 broth (Difco, Becton, Dickinson) supplemented with 2.5 ml of 20% Tween-80, 4 ml of 50% glycerol and 100 ml of OADC (7H9/Gly/TWEN/OADC). Uninoculated 7H9/Gly/TWEN/OADC was included as a negative control. The cultures were stored in air-tight containers, wrapped in an outer bag, and then incubated at 37°C. The cultures were checked for 14 days and those which presented the growth were prepared for genomic DNA extraction. Some cultures with poor growth were checked for more than three weeks, and DNA extraction was performed directly on MGIT for those that were not able to grow properly on 7H9/Gly/TWEN/OADC.

*Nucleic acid extraction by the CTAB method*

The bacteria in the media were poured into labelled 15 ml falcon tubes, then heat killed at 80°C for 1 hour before being removed from the BSL3, and genomic DNA (gDNA) extraction was performed using a standardised methodology: phenol-chloroform (CTAB; Hexadecyltrimethylammonium bromide) and sodium dodecyl sulfate (SDS) method. A NanoDropTM spectrophotometer (Thermo Fisher Scientific, Waltham, Massachusetts, USA) and Agarose gel electrophoresis were used to determine the nucleic acid quality and quantity control for individual samples. At the general laboratory (BSL2), the falcon tubes were centrifuged at 4000xg for 30 min at room temperature to discard the supernatant and the pellet was resuspended in 400 µl of TE buffer (Tris EDTA; pH8). To degrade the cell wall, 50 µl of lysozyme 10 mg/ml (50 mg Roche, Germany) was dispensed to each tube with bacterial suspension and incubated at 37°C overnight in the shaker oven. The next day, a solution comprising 70 µl of 10% SDS and 5 µl Proteinase K (10 mg/ml) was added, and tubes were incubated at 65 °C for 10 min. To separate cell debris and degraded proteins from the DNA, 100µl of 5M NaCl and 100 µl of pre-warmed (65°C) of CTAB|NaCl solution were subsequently added in each tube. The solution was vortexed until the solution became milky and incubated at 65 °C for 10 min, after which 750µl of chloroform/isoamyl alcohol (CI; 24:1, brought to RT) was used to suspend nucleic acid and help remove the CTABprotein/polysaccharide complexes with centrifugation at 4000 x g for 10 mins to separate the solution into 3 layers. The supernatant was collected and transferred into clean 2ml Eppendorf^TM^ tubes and was mixed with 750μl of ice-cold isopropanol and incubated at -20°C for at least 1 hour. The centrifugation step was repeated at room temperature for 30 mins, and the supernatant was carefully removed and discarded. The pellet was washed with 700μl of ice-cold 75% ethanol, centrifugation was repeated for 15 mins, and the supernatant was carefully removed and discarded.

The crude DNA was allowed to air dry for 48 hours in a biosafety cabinet class 2. Finally, the DNA was resuspended in 50 µl TE buffer (pH 8) and stored at 4°C. The DNA quality and quantity were assessed through a spectrophotometer and agarose gel electrophoresis. Only DNA samples respecting the following criteria were sent for whole genome sequencing: a minimum concentration of 20 ng/ µl, a 260/280 ratio at 1.8 (values between 1.79 and 2.0) and a 260/230 ratio > 1.8. if the sample had great DNA concentration but a critical ratio (260/280 and 260/230), the DNA cleaning protocol was implemented by washing the eluted DNA sample with a solution of 100% ethanol and 3M sodium acetate. For 50 µl of the DNA sample, 137.5 µl of 100% ethanol and 5 µl of 3M sodium acetate were added into the tube and mixed then incubated at -20°C for at least 2 hours. The mixture was spun at the maximum speed for 10-20 min at 4°C. 1 ml of 75% ethanol was dispensed in each tube after the supernatant was discarded. The centrifugation step was repeated at 4°C for 10-15 mins, and the supernatant was carefully removed. The DNA was resuspended in 50 µl TE buffer (pH 8) after complete evaporation of ethanol and stored at 4°C for quantity checking. The integrity of DNA was checked through 1% of the gel electrophoresis with 1X TAE buffer by running 1 µl of DNA with 5 µl of loading dye at 100V for 45 min.

*Whole Genome Sequencing (WGS)*

Briefly, short-read libraries were prepared using the Illumina Nextera XT DNA Library Preparation Kit (Illumina, Inc., San Diego, CA) or the CD Universal DNA Library Prep Kit for Illumina following the manufacturer’s protocol. After normalisation and pooling, the libraries were whole genome sequenced (paired-ended, barcoded, and multiplexed) on an Illumina HiSeq platform: Illumina HiSeq X Ten or MGI sequencer: MGI DNBSEQ-T7/DNBSEQ-G400. The depth coverage of the sequencing was 100x, and the Phred quality score Q30 > 85%.The resulting sequences were gathered with those stored in the server after quality control and used for whole genome sequence analysis. They were combined with basic demographics for genomic, phylogeny and statistical analysis at the TB Genomics unit of Molecular Biology and Human Genetics division at Stellenbosch University (SU).

*Phylogeny tree construction*

The FASTA format from the MTBseq analysis was used for phylogenomic analysis. The resulting multi-sequence alignment (MSA) file was cleaned with SNP-sites v2.5.1 (1). If an alignment contained a group of more than two identical sequences, we excluded all the groups. The MSA file was converted into the nexus format and used as input for Maximum-likelihood topologies using IQ-TREE2 v2.2.0.3 (<http://www.iqtree.org/>) (2). We determined the best-fit models by using ModelFinder (3) with the “-m MFP” argument. Models were chosen as follows: TVM+F+ASC+R2. Trees were rooted with the reference genome *Mycobacterium. tuberculosis* H37Rv (version NC_000962.3) as an outgroup. We assessed the robustness of the inferred phylogenetic tree with ultrafast bootstrap (UFBoot) for 1000 replicates (4) and the support of the specific branches within the trees using an approximate likelihood ratio test (SH-aLRT) for 1000 replicates with “-alrt” argument (5). In order to avoid severe model violations, we appended the “-bnni” argument to optimise UFBoot by nearest neighbour interchange. We visualised the evolutionary relationships between genotypes by using iTOL(6).

*Statistical analysis*

Logistic regression models were used for univariate and multivariate analysis to assess associations between each risk factor and lineage, cluster status (belonging to a cluster or not) and cluster size. For lineage, three dichotomous variables were created for samples belonging to a lineage, or not, e.g. lineage 4 and not lineage 4 (all other lineages combined: lineage 2.2.1 and lineage 2.2.2). These variables were the dependent variables in models with the risk factors as independent variables, one at a time for the univariate analysis, and including all the risk factors reported in Supplementary Table 4, for the multivariate analysis.

Similarly, the dependent variables for the cluster status and cluster size were dichotomous variables, with one category compared to the other categories combined. The independent variables were as described above for the lineage models. To assess multicollinearity, we calculated variance inflation factors (VIFs) using “estat vif command after fitting the model.

We conducted a risk factor analysis for cluster and cluster size defined at the cutoff SNP=0, SNP≤5 and SNP≤12. First, we compared the XDR-TB strain characteristics between “clustered” and “non-clustered” isolates to identify the risk factors to propagate. Second, we compared the characteristics of “small clusters”, “large clusters”, and “very large clusters” to identify the risk factors for each specific size of the cluster that is associated with more spreading potential of the disease in the community

**Supplementary Results**

### ***Genotypic Drug Resistance (gDST) Profile***

*Pyrazinamide resistance variants*

In this cohort, 506 isolates exhibited twenty-eight distinct *pncA* variants, with some variants appearing in combination (**Table S4**). The two most prevalent variants occurred only in sublineage 2.2.2 isolates: c.517dupG (n=174/590; 29.49%) and p.C14R (n=151/590; 25.59%). In contrast to sublineage 2.2.2, which harboured only 4 different variants, sublineage 2.2.1 strains harboured a variety of mutations at frequencies ranging from one isolate (<1%) to 43 (7.29%) where the most observed were p.D8N (n=43/590; 7.29%), p.T100I+T160A (n=19/590; 3.22%,), p.Y103* (n=19/590; 3.22%), p.T135P (n=14/590; 2.37%), p.S164P (n=8/590; 1.36%) and p.V130A (n=8/590; 1.36%) variants. Lineage 4 strains harboured a similar variety of mutations with the p.V139M (n=26/590; 4.41%) and c.456dupC (n=9/590; 1.53%) variants were mostly found.

*Ethambutol resistance variants*

Ethambutol resistance was characterised by variants within the *embA* and *embB* genes in 98.14% (n=579/590) of isolates (**Table S4**). The most prevalent ethambutol-resistance variants were p.M306I (n=355/590; 60.16%) and p.M306V (n=201/590; 34.06%). However, the p.M306I variant was mostly found only in sublineage 2.2.2 strains, while the p.M306V variant was predominantly observed in sublineage 2.2.1 and lineage 4 strains. Seven additional variants across the two genes accounted for resistance in 23 isolates.

*Streptomycin resistance variants*

Resistance variants within the *gid*, *rpsL*, and *rrs* genes were observed in 80.17% (n=473/590) of XDR-TB isolates (**Table S4**). The most common variant in *gid* was L79S (n=320/590; 54.24%), which always occurred with *rrs*_n.514A>C and only in sublineage 2.2.2 isolates. The *rpsL*_p.K43R variant (n=100/590; 16.95%) was mostly observed in sublineage 2.2.1 and lineage 4 strains, while the *rrs*_n.514A>C variant alone (n=349/590; 59.15%) was only present in sublineage 2.2.1 (n=25) and sublineage 2.2.2 (n=323) strains.

*Ethionamide resistance variants*

The ethionamide resistance variables were identified within the *ethA*, *ethR*, *fabG1* and *inhA* genes (**Table S4**). Variants in the *fabG1/inhA* promoter were the most frequently observed cause of ETH resistance (n=344/590; 58.31%). Eleven different *ethA* mutations were observed in 411 isolates (69.66%), with or without additional ETH resistance variants, and each was unique to one (sub) lineage. The most common of these was *ethA_*p.A381P, occurring in 337 sublineage 2.2.2 isolates (57.11%). Only one *ethR* variant (p.A95T) was observed in 145 sublineage 2.2.1 isolates (24.57%) and always in conjunction with at least one other ETH resistance-conferring variant. Four different *inhA* gene mutations were present in 104 isolates (17.62%), but only p.S94A (n=3/590; 0.51%) occurred without additional ETH resistance variants.

*Para-aminosalicylic acid resistance variants*

Variants that confer PAS resistance were identified in 2.71% (n=16/590) of isolates (**Table S4**). These variants were found within the *folC* (43.75%, n=7/16), *thyA* (25%, n=4/16) and *thyX* (31.25%, n=5/16) genes. The variants were mostly found in sublineage 2.2.2 strains.

**Genomic Clustering Analysis**

***Clusters and size***

*5 SNP threshold*

At the 5 SNP threshold (recent transmission), strains were grouped into 35 genomic clusters (**Supplementary Table S6)**, containing 2 to 9, 12, 24, 84, 126 and 149 isolates. Clusters of 2 isolates was the most common, with 13 groups of isolates, followed by cluster sizes 3, 4 and 5, with 8, 3 and 3 groups of isolates, respectively (**Supplementary Table S6)**. Sublineage 2.2.1 strains formed more small clusters than sublineage 2.2.2 and lineage 4 strains. However, sublineage 2.2.2 strains clustered more into larger groups (with sizes of 126 and 149 isolates) than isolates from sublineage 2.2.1 (with sizes of 24 and 84 isolates). The distribution of the clusters over the study period reveals that the majority were located with a period of less than or equal to 3 years (**Figure 4**).

*12 SNP threshold*

With the 12 SNP threshold (SNP≤12), strains were grouped into 17 genomic clusters containing 2 to 9, 26, 27, 142, 146 and 171 isolates (**Supplementary Table S6**).

**Logistic regression analysis**

***Factors associated with genomic clustering***

*5 SNP threshold*

In the univariate analysis, we found that clustered isolates, compared to unclustered group were more likely to belong to sublineage 2.2.2 (OR=13.47; p<0.0001) and sublineage 2.2.1 (OR=4.96; p<0.0001), and originated from patients living within Garden Route (OR=3.45; p=0.043) and Cape Town Metropole (OR=3.07; p=0.013) HCDs. In the multivariate analysis, isolates belonging to sublineage 2.2.2 (OR=22.02; p<0.0001) and sublineage 2.2.1 (OR=7.96; p<0.0001) and isolated in 2018 (OR=8.81; p=0.014) were more likely to cluster(**Supplementary Table S7**).

***Factors associated with genomic cluster size***

*Threshold 5 SNP*

*Very large cluster size*

In univariate analysis, isolates within very large clusters, compared to other cluster sizes (small and large) were more likely to be collected in the years 2010 (OR=3.84; p=0.007), 2011 (OR=2.62; p=0.049), 2012 (OR=3.72; p=0.007) and 2016 (OR=3.16; p=0.025). Isolates in very large clusters were also more likely to originate from patients living within Cape Town Metropole (OR=9.81; p<0.0001), Garden Route (OR=8.66; p=0.001) and Cape Winelands (OR=4.39; p=0.024) HCDs (**Supplementary Table S9**). These isolates were less likely to belong to sublineage 2.2.1 (OR=0.14; p<0.0001).

In the multivariate analysis, isolates within very large clusters compared to other cluster sizes (small and large) were more likely to be collected in the years 2010 (OR=4.21; p=0.016) and 2016 (OR=3.73; p=0.034), originated from patients living within Cape Town Metropole (OR=6.67; p<0.0001), Garden Route (OR=4.11; p=0.037) and Cape Winelands (OR=3.99; p=0.025) HCDs. These isolates were also less likely to belong to sublineage 2.2.1 (OR=0.23; p<0.0001).

*Large cluster size*

In the univariate analysis, isolates within large clusters, compared to other cluster sizes (small and very large) were less likely to be isolated from patients aged between 40 and 49 years old (OR=0.42; p=0.045) and in patients living within Cape Town Metropole (OR=0.05; p<0.0001) Cape Winelands (OR=0.07; p=0.024) and Garden Route (OR=0.09; p=0.001) HCDs. However, these isolates were more likely to belong to sublineage 2.2.1 (OR=3.92; p<0.0001).

In the multivariable analysis, isolates within the large clusters, compared to other cluster sizes (small and very large) were more likely to belong to sublineage 2.2.1 strains (OR=1.95; p=0.046) and less likely to be found in patients living within Cape Town Metropole (OR=0.07; p<0.0001) Cape Winelands (OR=0.12; p=0.003) and Garden Route (OR=0.18; p=0.012) HCDs.

*Small cluster size*

Isolates within the small clusters, compared to other cluster sizes (large and very large), were less likely to be isolated in the years 2010 (OR=0.05; p=0.007) and 2012 (OR=0.04; p=0.006), and more likely to belong to sublineage 2.2.1 (OR=6.42; p<0.0001) in univariate analysis.

In the multivariable analysis, isolates within small cluster sizes, compared to other cluster sizes (large and very large), were less likely to be identified in the years 2010 (OR=0.03; p=0.007) and 2012 (OR=0.07; p=0.024). These isolates were also more likely to belong to sublineage 2.2.1 (OR=5.65; p<0.0001).

**Supplementary results:**


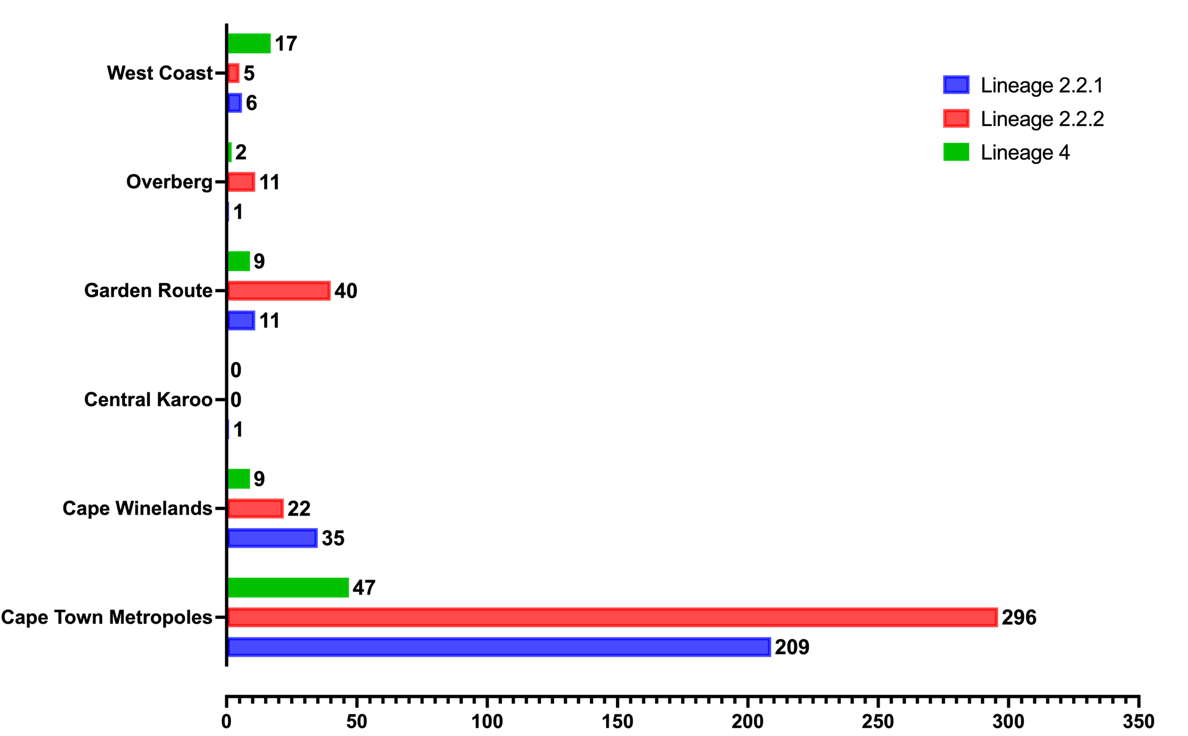


**Figure 1:** Distribution of XDR-TB isolates lineages per Healthcare district in Western Cape Province between 2010 and 2019.


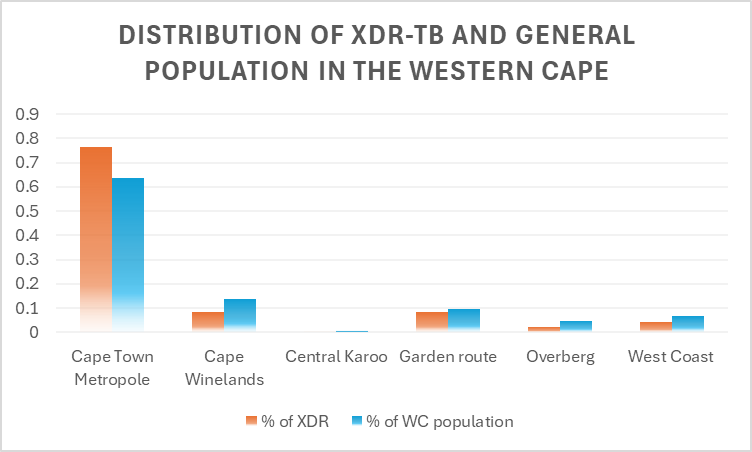


**Figure 2:** Distribution of XDR-TB and the general population in the Western Cape (Year 2016). https://en.wikipedia.org/wiki/Western_Cape#Municipalities

**Figure 3:** Distribution of clustered strains per SNP distance threshold. The proportion of clustered vs unclustered strains changes according to the SNP distance threshold applied.

**Figure 4:** Timeline for genomic clusters of XDR-TB isolates per genotypes at 5 SNP threshold distance.

**Figure 5:** Maximum-likelihood phylogeny of 694 pre-XDR and XDR *mycobacterium tuberculosis* isolates. The colour-coded annotation includes lineages, sublineages and drug-resistance profiles. The colour of the branches indicates the different degrees of bootstrap.

1. Page AJ, Taylor B, Delaney AJ, Soares J, Seemann T, Keane JA, et al. SNP-sites: rapid efficient extraction of SNPs from multi-FASTA alignments. Microb Genom. 2016 Apr;2(4):e000056.

2. Minh BQ, Schmidt HA, Chernomor O, Schrempf D, Woodhams MD, von Haeseler A, et al. IQ-TREE 2: New Models and Efficient Methods for Phylogenetic Inference in the Genomic Era. Molecular Biology and Evolution [Internet]. 2020 May 1 [cited 2023 Jul 20];37(5):1530–4. Available from: https://doi.org/10.1093/molbev/msaa015

3. Kalyaanamoorthy S, Minh BQ, Wong TKF, Von Haeseler A, Jermiin LS. ModelFinder: fast model selection for accurate phylogenetic estimates. Nat Methods [Internet]. 2017 Jun [cited 2023 Jul 20];14(6):587–9. Available from: https://www.nature.com/articles/nmeth.4285

4. Hoang DT, Chernomor O, von Haeseler A, Minh BQ, Vinh LS. UFBoot2: Improving the Ultrafast Bootstrap Approximation. Molecular Biology and Evolution [Internet]. 2018 Feb 1 [cited 2023 Jul 20];35(2):518–22. Available from: https://doi.org/10.1093/molbev/msx281

5. Guindon S, Dufayard JF, Lefort V, Anisimova M, Hordijk W, Gascuel O. New Algorithms and Methods to Estimate Maximum-Likelihood Phylogenies: Assessing the Performance of PhyML 3.0. Systematic Biology [Internet]. 2010 May 1 [cited 2023 Jul 20];59(3):307–21. Available from: https://doi.org/10.1093/sysbio/syq010

6. Letunic I, Bork P. Interactive Tree Of Life (iTOL) v5: an online tool for phylogenetic tree display and annotation. Nucleic Acids Research [Internet]. 2021 Jul 2 [cited 2023 Aug 8];49(W1):W293–6. Available from: https://doi.org/10.1093/nar/gkab301
